# Dynamic of cold-atom tips in anharmonic potentials

**DOI:** 10.3762/bjnano.7.148

**Published:** 2016-10-31

**Authors:** Tobias Menold, Peter Federsel, Carola Rogulj, Hendrik Hölscher, József Fortágh, Andreas Günther

**Affiliations:** 1 Physikalisches Institut, Eberhardt-Karls-Universität Tübingen, D-72076 Tübingen, Germany; 2 Institut für Mikrostrukturtechnik, Karlsruher Institut für Technologie, 76344 Eggenstein-Leopoldshafen, Germany

**Keywords:** anharmonic motion, cold-atom scanning probe microscopy, dephasing, dynamic mode, tip oscillation

## Abstract

**Background:** Understanding the dynamics of ultracold quantum gases in an anharmonic potential is essential for applications in the new field of cold-atom scanning probe microscopy. Therein, cold atomic ensembles are used as sensitive probe tips to investigate nanostructured surfaces and surface-near potentials, which typically cause anharmonic tip motion.

**Results:** Besides a theoretical description of this anharmonic tip motion, we introduce a novel method for detecting the cold-atom tip dynamics in situ and real time. In agreement with theory, the first measurements show that particle interactions and anharmonic motion have a significant impact on the tip dynamics.

**Conclusion:** Our findings will be crucial for the realization of high-sensitivity force spectroscopy with cold-atom tips and could possibly allow for the development of advanced spectroscopic techniques such as Q-control.

## Introduction

The development of novel scanning probe techniques has lead to tremendous improvements in investigating nanomaterials [1]. Starting with conventional force and tunneling microscopes [2–3], various methods have emerged for detecting topographic [4], electromagnetic [5–6], thermal [7] and even chemical properties [8–9] of matter. At the same time, the research field of quantum atom optics provided access to new “quantum matter” [10–11]. Since then, ultracold atoms have been used for studying multiple many-body effects, ranging from Mott-insulator transitions [12] to Feshbach [13] and Efimov [14] resonances. Preparing and manipulating these quantum gases in the direct vicinity of micro- and nanostructured surfaces [15–22] paved the way to cold-atom surface probing [23–28] and finally allowed for the realization of cold-atom scanning probe microscopy [29–31]. Here, an ultracold cloud of atoms is used as sensitive probe tip in a scanning microscope. First realizations have demonstrated this to be suitable for topographic [29] and dispersion force measurements [30]. Thereby, the basic principles of force microscopy have been transferred to cold atoms, including a dynamic operation mode [29]. Here, the cold-atom tip oscillates with respect to the surface of interest and information is extracted from measuring the position-dependent oscillation amplitude and frequency. Precision force spectroscopy [32] with cold atoms comes thus into direct reach, with expected force sensitivities in the yN-regime [29]. Therefore, the dynamics of cold-atom tips in an anharmonic potential must be fully understood and a method for real-time observation of the tip motion must be developed.

In this manuscript, we experimentally and theoretically study the dynamic motion of an oscillating cold-atom tip in an anharmonic potential. The dynamic is shown to be completely different from conventional solid state tips, which are typically treated as rigid bodies. For the cold-atom tip, the situation is much more complex as the tip behaves like a thermal gas of atoms, best described via a distribution function. We show that collisions between particles and the anharmonicity of the potential will have a strong influence on the overall tip dynamics. In addition, we introduce a novel method for local density probing, allowing detection of the tip dynamics in real time. The first measurements on oscillating cold-atom tips show very good agreement with theoretical and numerical calculations. Our findings will be essential for future force spectroscopy with cold atoms and could possibly allow for active feedback control of the tip motion. Methods like Q-control [33–34], which have been very successful in conventional force microscopy [35–36], are therefore realizable.

The article is structured as follows: We start by describing the theory of tip motion in harmonic and anharmonic potentials. Using analytic expressions and numerical calculations, we describe the expected tip motion, including particle dephasing and collision effects. In the following section, we introduce a specific real-time observation scheme of the tip motion, which is based on sensitive single atom detection. We analyze the expected detection signal for harmonic and anharmonic tip motion. In the experimental section we present results on oscillating tip measurements and compare them to theory. Special attention is given to the effects of particle dephasing and collisions. The manuscript closes with a conclusion and a methods section, describing the details of the numerical simulations.

## Theory of tip dynamics

The dynamics of a cold-atom tip in an anharmonic potential is fundamentally different from the dynamics of a conventional solid-state tip. While the solid tip behaves like a rigid nondeformable body and can be described by its position 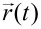 and momentum 
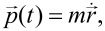
 the cold-atom tip is a weakly interacting gas of thermal atoms. It is characterized by the position 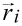 and velocity 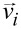 of the individual particles *i* = 1…*N*. To simplify the problem, thermal atomic ensembles are typically described by their phase-space distribution function 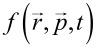 defining the probability for an atom to be found at position 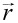 and momentum 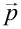. Similar to the equation of motion, a partial differential equation (PDE) can then be found from Liouville’s theorem, characterizing the cloud’s dynamics in phase space [37]

[1]
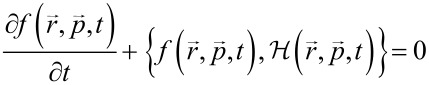


with the classical Hamiltonian 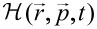 and the Poisson brackets {}. If particle interactions come into play, the dynamic becomes even more complex. Liouville’s theorem must then be replaced by the Boltzmann kinetic equation [37], which adds an additional collision integral 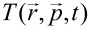 to the right hand side of Equation 1. At ultracold temperatures in the μK regime, this integral accounts for s-wave scattering processes, with a cross section σ given by the s-wave scattering length *a*_0_, which is 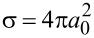 for distinguishable and 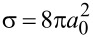 for indistinguishable particles. Deriving an analytic solution to Equation 1 is typically nontrivial; therefore, numerical solutions are often required.

If the cold-atom tip is cooled further, a Bose–Einstein condensate is created and the tip shows quantum behavior [38–39]. In this case the quantum tip behaves like a superfluid and is typically described by a quantum mechanical wave function 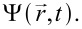 The tip dynamic is then found by solving the corresponding Schroedinger equation. Particle interactions can be taken into account via a mean field approach, yielding the so called Gross–Pitaevskii equation [40]. In this work, however, we restrict the discussion to the dynamics of a pure cold-atom tip, described by a thermal gas of atoms.

### Dynamics in harmonic potentials

We start by analyzing the dynamics of a cold-atom tip in a harmonic potential. Therefore, we describe the tip as an ideal gas of noninteracting particles that are in thermal equilibrium. Each particle is exposed to the same external potential, which we assume to be one-dimensional

[2]
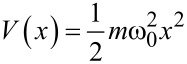


with *m* being the particle mass and ω_0_ the resonance frequency. Such harmonic potentials are typically found in the center of magnetic or optical traps, which are used to confine ultracold atoms in an ultrahigh vacuum environment. This assures lifetimes of the cold-atom tip in the 100 s regime, as collisions with background atoms are strongly reduced.

For a single particle *i*, the dynamics in such a harmonic potential is determined from the equation of motion and the starting conditions *x_i0_* = *x_i_*(0) and 
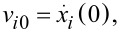
 resulting in periodic particle oscillations

[3]
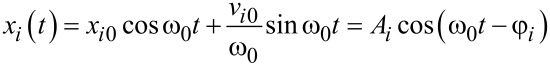


with oscillation amplitudes 
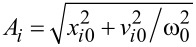
 and phases φ*_i_* = arctan(*v_i0_*/(ω_0_*x_i0_*)). Independent of the start energy, all particles oscillate at the same frequency and relative phases are conserved.

The full dynamics of the cold-atom tip, however, is best understood by means of its phase-space distribution 
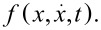
 Following Liouville’s theorem from Equation 1 and using 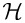 = *p*^2^/2*m* + *V*(*x*), the tip dynamics is characterized by

[4]
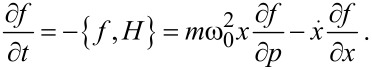


Assuming a thermalized cold-atom tip at temperature *T*, which is displaced at time *t* = 0 by an amount *A* from its equilibrium position, the solution to Equation 4 becomes

[5]
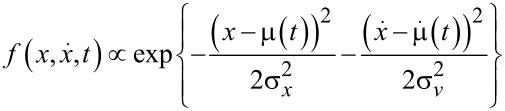


with μ(*t*) = *A*cosω_0_*t*, σ*_x_* = σ*_v_*/ω_0_ and 
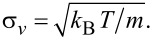
 The shape of the cold-atom tip is then given by the spatial density distribution

[6]
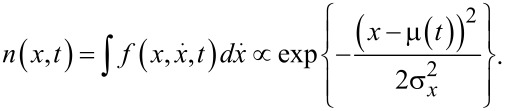


At all times, the cold-atom tip shows a Gaussian shape with constant width σ*_x_*. The dynamics is thus fully included in the center-of-mass oscillation μ(*t*). In this sense, a cold-atom tip oscillating in a harmonic potential behaves very much like a solid tip in an atomic force microscope.

**Figure 1:**
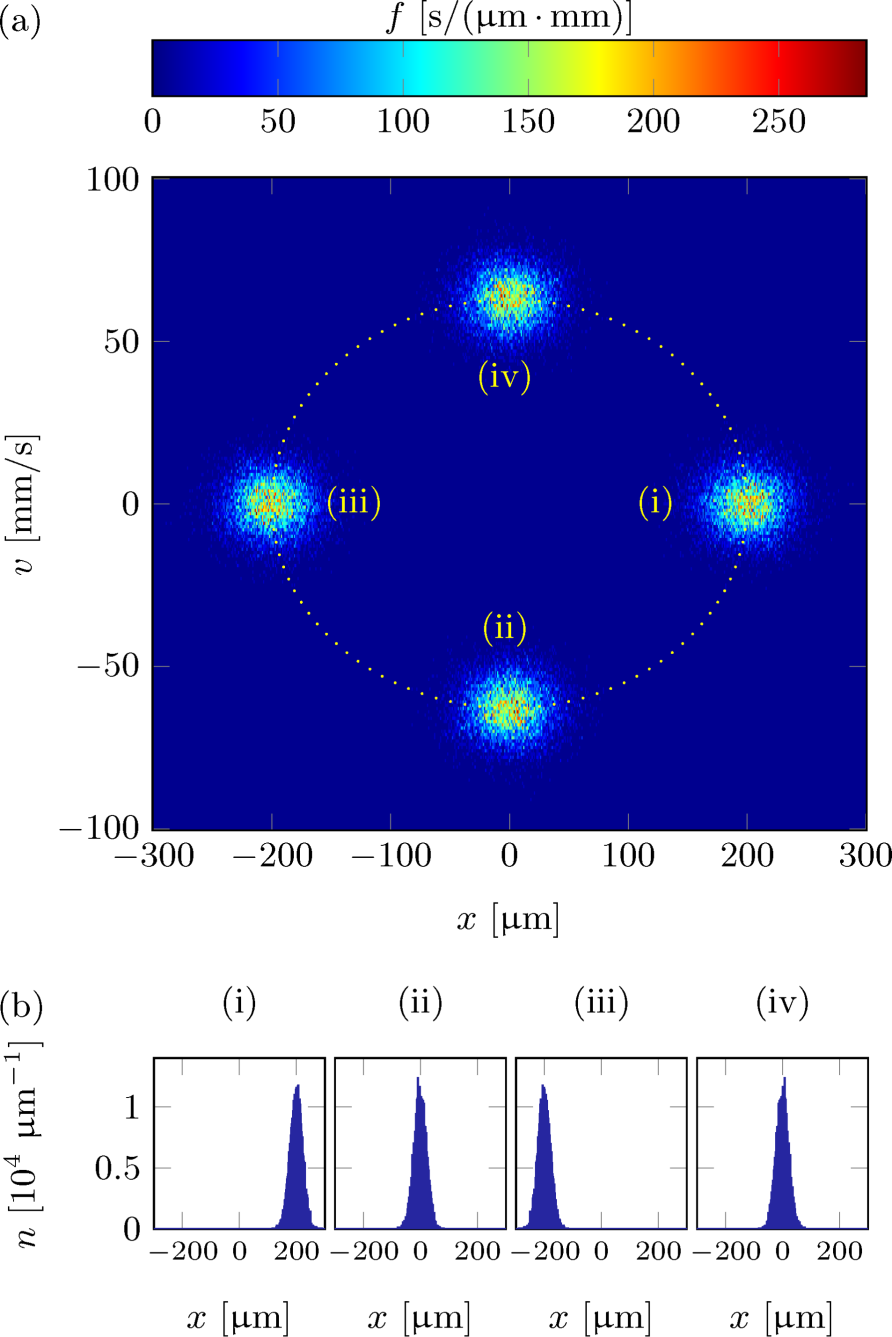
Numerical simulation of an oscillating cold-atom tip in a harmonic potential of fundamental period *T* = 1/(50 Hz): (a) phase-space density and (b) density profiles at *t* = 0 T, 2.25 T, 4.5 T, 6.75 T (i–iv) after the initial tip displacement. The simulations do not change if particle collisions are taken into account.

To illustrate the dynamics of the cold-atom tip, Figure 1a shows 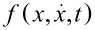 in the two-dimensional phase space at four different times after the initial displacement. The data are derived from a numerical simulation of the cold atom tip with 5 × 10^5^ atoms at 500 nK, moving in an harmonic potential with ω_0_ = 2π × 50 Hz and an initial displacement of *A* = 200 μm.

Due to Liouville’s theorem, the phase-space volume of the atomic cloud is conserved at all times. As all particles oscillate through the phase space with the same frequency ω_0_, the shape of *f* is also conserved. The spatial density distribution is then found by integrating the phase-space distribution along the velocity axis. For the situation in Figure 1a, it becomes immediately clear that the tip shape remains constant and the dynamics is only due to a center-of-mass oscillation with frequency ω_0_. Figure 1b shows the density profiles of the probe tip as extracted from the phase space densities in Figure 1a.

The situation remains unchanged even if particle interactions are taken into account. As the curvature of the potential is constant in space, the cloud is always in thermal equilibrium, such that collisions will not affect the dynamics of the cloud in phase space. This will dramatically change in anharmonic potentials, as discussed in the next section.

### Dynamics in anharmonic potentials

To understand the motion of cold-atom tips in an anharmonic potential, the nonlinear motion effects that occur if a classical point mass is oscillating in a cubic oscillator are implemented. Here, the potential is given by

[7]
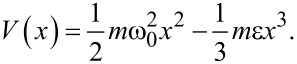


with ε describing the strength of the anharmonicity. The corresponding equation of motion is given by

[8]
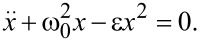


Assuming the starting conditions *x*(0) = *x*_0_ and 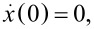 this differential equation can be solved in the limit 
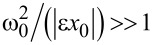
 using second order perturbation theory in combination with the Poincaré–Lindstedt method to avoid secular terms [41]. With α*_i_* = α*_i_*(*x*_0_,ω_0_,ε) being the contributions of the base frequency (*i* = 1) and higher harmonics (*i >* 1) to the particle dynamics [41–42], the solution reads

[9]
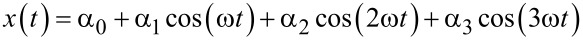


which is a periodic function in time with the fundamental frequency ω depending on the initial displacement [42].

[10]
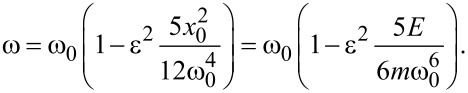


For arbitrary starting conditions the oscillation frequency in an anharmonic potential will thus depend on the total initial energy *E* (kinetic and potential energy) of the particle. At the same time, the spectrum of the oscillation will contain not only the fundamental frequency but also higher harmonics (see Equation 9).

**Figure 2:**
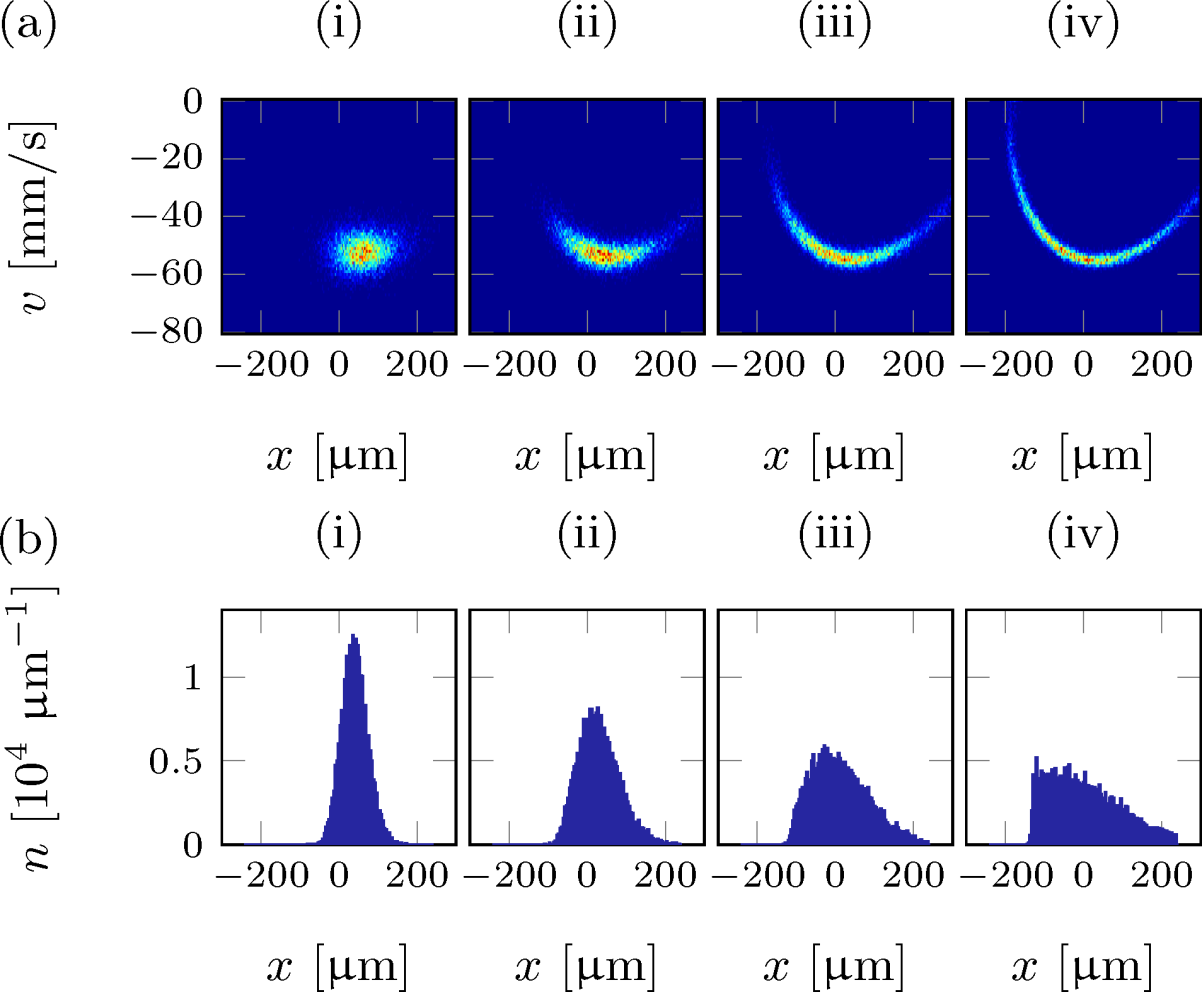
Numerical simulation of an oscillating cold-atom tip in an anharmonic potential of fundamental period *T* = 1/(50 Hz) and anharmonicity ε = 2 × 10^8^ m^−1^ s^−2^: (a) phase-space density and (b) density profiles at times *t* = 0.25 T, 2.25 T, 4.25 T, 6.25 T (i–iv) after the initial tip displacement. Particle interactions (collisions) are not taken into account.

Following the above considerations and neglecting particle interactions, the dynamics of a cold-atom tip in an anharmonic potential can be understood as superposition of all single particle oscillations. Figure 2 illustrates the resulting tip dynamics in the two-dimensional phase space.

The tip parameters have been chosen as before (*T* = 500 nK, *N* = 5 × 10^5^, ω_0_ = 2π × 50 Hz) with an anharmonicity ε = 2 × 10^8^ m^−1^ s^−2^. Figure 2a shows the distribution function 
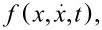
 as derived from a full numerical simulation at four different times after the initial displacement of *A* = 200 μm. Figure 2b shows the corresponding density profiles. Depending on the initial energy, each particle follows its own phase-space trajectory and oscillates clockwise around the origin with its own fundamental frequency. Low energetic particles will follow trajectories closer to the phase space origin and oscillate faster than particles with higher energies. Right after the initial displacement of the cold-atom tip, all particles will start their oscillation in phase, resulting in a narrow distribution function and a clear center-of-mass oscillation (i). As time goes by, the higher energetic particles will drag more and more behind, leading to a relative dephasing between particle oscillations (ii–iv). This results in a spread of the phase-space distribution and a center-of-mass shift towards the equilibrium position. For the density distribution, the dephasing will thus lead to a broadening of the distribution function, a decrease of the peak density and a damping of the center-of-mass oscillation. Finally, the atoms will be spread over the whole oscillation region, with no center-of-mass oscillation remaining. The density distribution will then become static. Following Equation 10, the timescale τ*_d_* of the dephasing is given by the relative spread of the fundamental oscillation frequencies

[11]
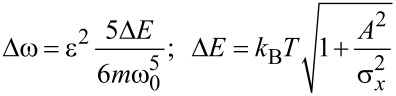


with the energy spread Δ*E* calculated for a Gaussian start distribution with initial displacement *A*. Dephasing will then occur on a timescale

[12]
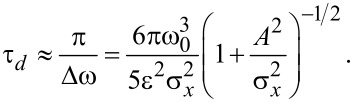


Atom tips at lower temperatures, smaller displacements, and less anharmonic potential will then lead to increased dephasing times. However, even for small oscillation amplitudes, Equation 12 sets an upper limit to the dephasing time

[13]
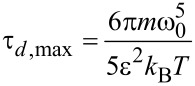


depending only on the tip temperature and anharmonicity of the trap.

**Figure 3:**
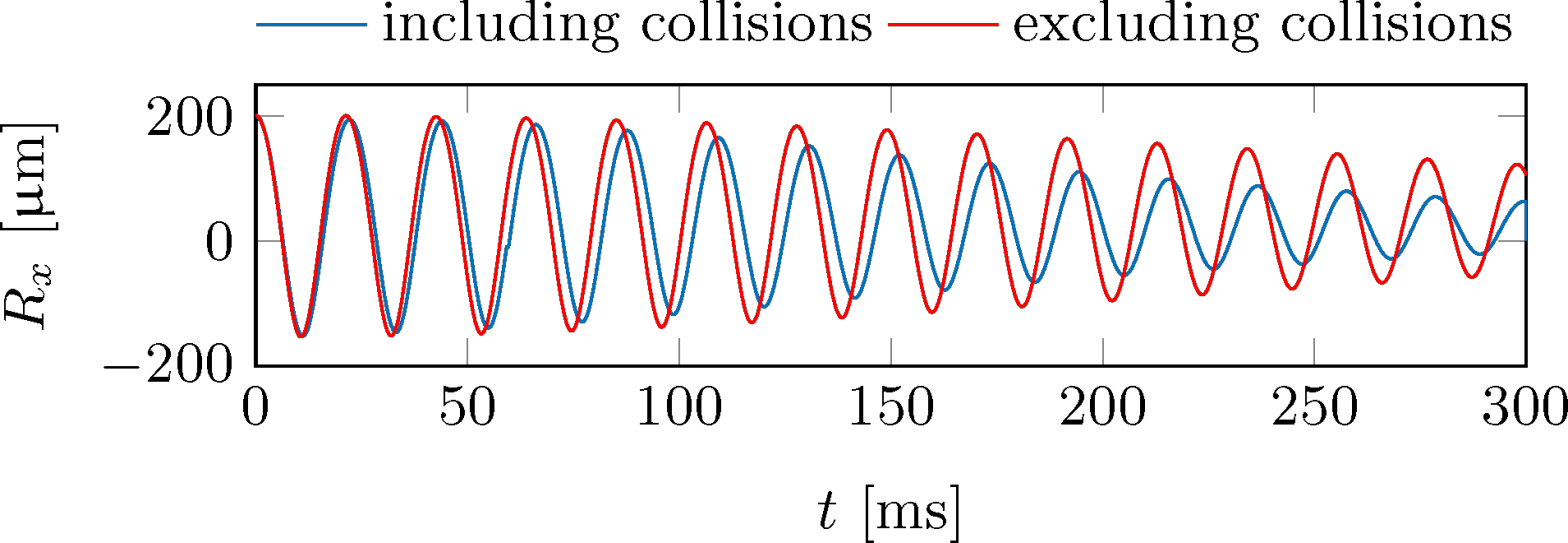
Center-of-mass oscillation of a cold-atom tip in an anharmonic potential with/without particle collisions (blue/red solid line). The simulation parameters have been chosen as before: *T* = 500 nK, *N* = 5 × 10^5^ atoms, ε = 2 × 10^8^ m^−1^ s^−2^, (ω*_x_*,ω*_y_*,ω*_z_*) = 2π × (50, 500, 500) Hz, *A* = 200 μm.

Collisions between particles will only have to be taken into account in 2D and 3D systems, where energy can be transferred between the different spatial directions. Collisions will then lead to a frequent redistribution of energy among the particles, leading to heating in the transverse directions. Therefore, the center-of-mass motion is damped even stronger as in the case of noninteracting particles. Figure 3 shows the resulting center-of-mass motion of an oscillating cold-atom tip (blue solid line), as derived from a 3D numerical calculation, which includes particle collisions.

The simulation parameters are identical as before, while the anharmonic trap has been extended in the transversal direction by a harmonic confinement with frequency ω*_y/z_* = 2π × 500 Hz. For comparison, Figure 3 includes the result for noninteracting particles, showing a reduced damping. Following Equation 12, the damping time is 660 ms.

## Detecting tip dynamics

The detection of a cold-atom tip is fundamentally different from conventional scanning probe techniques. In an atomic force microscope, for instance, the tip position can be monitored in real-time using methods like laser beam deflection [43–44], laser interferometry [45–46] or self-sensing [47–49]. For cold-atomic ensembles, the standard detection method is absorption imaging [50]. Here, the atoms are illuminated with coherent light, and a shadow image of the cloud is recorded on a CCD chip. This yields a two-dimensional density profile of the cold-atom tip, integrated along the direction of imaging. Typically, the cloud can be released from the trap before imaging, which allows for an additional ballistic expansion during the time of flight. For long expansion times, the absorption image yields the two-dimensional velocity distribution of the cold-atom tip. Assuming certain symmetries, the complete phase-space distribution function can then be obtained from a time-of-flight image series. Unfortunately, absorption imaging is a fully destructive process, such that the cold-atom tip is destroyed due to energy transfer from absorbed photons. At ultracold temperatures, the absorption of a single photon is sufficient to remove the corresponding atom from the tip. This was the main limitation in the first realization of a cold-atom scanning probe microscope [29], as new cold-atom tips could only be generated on timescales of about 60 s. The measurement time was thus orders of magnitude larger than in conventional scanning probe techniques.

Just recently, however, we have developed a new detection technique, which allows for local probing of the density distribution of a cold-atom tip in real time [51]. Therefore, a weak beam of atoms is outcoupled from the tip via microwave radiation and detected with multiphoton ionization and subsequent ion detection. Using this scheme, the outcoupling can be measured with high-temporal resolution and single-atom sensitivity. The outcoupling position can be precisely tuned via a microwave frequency, such that the outcoupling rate is a direct measure for the tip’s local density. This way, the density profile of a cold-atom tip at rest could be measured in situ with negligible atom losses [51].

Here, we extend the scheme to measure the dynamics of a cold-atom tip. Therefore we keep the outcoupling position 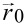 fixed in space and monitor the time-dependent ionization rate *Γ(t)* at the ion detector.

[14]
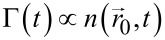


Measuring Γ(*t*) will then unveil dynamics within the distribution function.

### Detecting harmonic tip oscillations

For harmonic potentials the time-dependent density distribution of an oscillating tip is given via Equation 6, resulting in a time-dependent detector signal

[15]
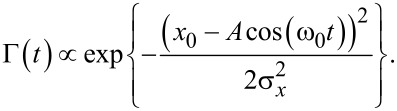


As a periodic function, it can be expanded to harmonic oscillations at multiples of the fundamental frequency ω_0_.

[16]
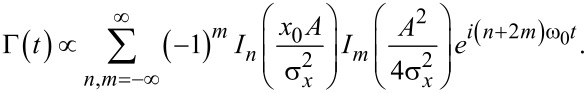


Here we used the Jakobi–Anger expansion with *I*_α_ being the modified Bessel functions of first kind. In the limit of small oscillation amplitudes *A <<* σ*_x_*, the leading terms in this expansion become

**Figure 4:**
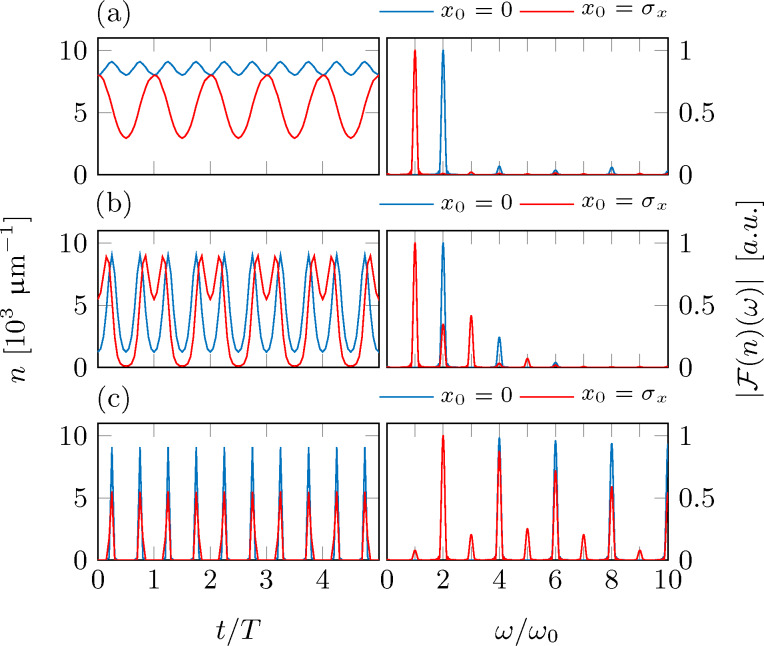
Simulated detection signal and its Fourier transform for a detector based on local density probing, as derived from a numerical simulation of a cold atom tip (*N* = 5 × 10^5^, *T* = 500 nK) oscillating in an harmonic potential (ω_0_ = 2π × 50 Hz). The oscillation amplitudes *A* have been set to (a) 0.5σ*_x_*, (b) 2σ*_x_* and (c) 200 μm. The outcoupling positions *x*_0_ are fixed to 0 (blue solid lines) and σ*_x_* (red solid lines).

[17]
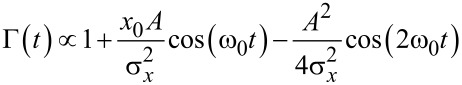


showing, that the count rate is in lowest order modulated at frequencies ω_0_ and 2ω_0_. The specific form of the detector signal will depend strongly on the outcoupling position *x*_0_. For |*x*_0_| *>> A* the detector signal is dominated by the fundamental oscillation frequency ω_0_. For |*x*_0_| *<< A*, however, this frequency component becomes negligible and the detector signal shows a modulation at twice the tip frequency. For *x*_0_ = 0, the modulation at frequency ω_0_ vanishes completely. Figure 4a shows the expected count rates and their Fourier spectra for two different outcoupling positions *x*_0_ = 0 and *x*_0_ = σ*_x_* for a small oscillation amplitude *A* = 0.5σ*_x_*.

The results are well described by the approximation from Equation 17. This changes for larger oscillation amplitudes, where higher harmonics must be taken into account as shown in Figure 4b for a cloud with oscillation amplitude *A* = 2σ. Increasing the oscillation amplitude further *A* = 200 μm ≈ 9σ*_x_*, more and more higher harmonics appear (Figure 4c).

### Detecting anharmonic tip oscillations

**Figure 5:**
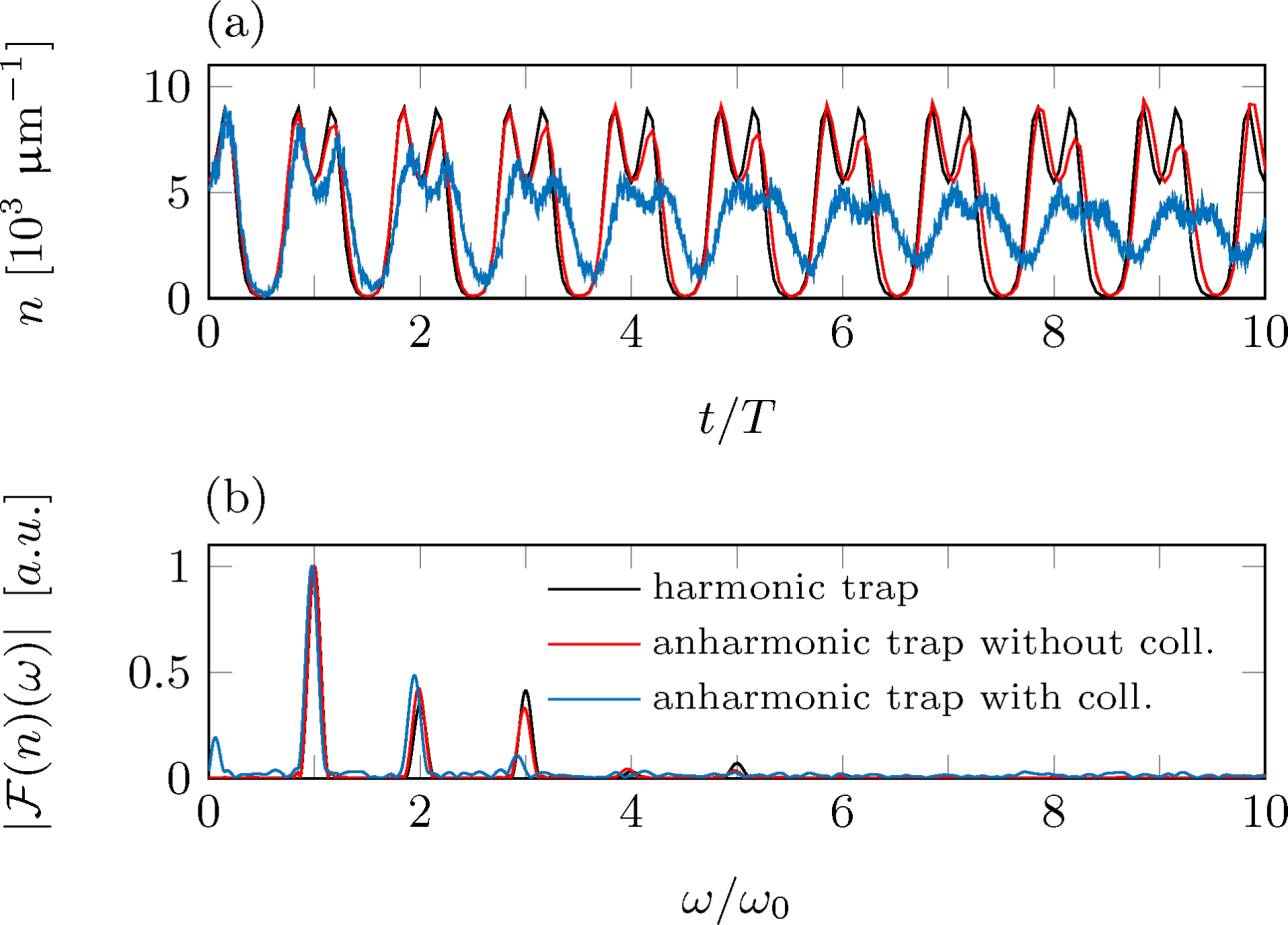
Simulated detection signal (a) and its Fourier transform (b) for a detector based on local density probing, as derived from a numerical simulation (*N* = 5 × 10^5^, *T* = 500 nK). The tip oscillates in a harmonic/anharmonic trap (black lines/colored lines) with ω_0_ = 2π × 50 Hz and ε = 2 × 10^8^ m^−1^ s^−2^. Particle collisions are taken into account (blue lines) or neglected (red lines). The oscillation amplitude and the outcoupling position have been fixed to *A* = 2σ*_x_* and *x*_0_ = σ*_x_*.

In the case of anharmonic potentials, the density distribution shows an oscillation, which seems to be damped due to dephasing of different particle trajectories. Starting with a well-localized cloud, it spreads more and more over the whole oscillation region. As the detector probes the density of the cloud at a fixed position, the detector signal will show clear signatures of a damped oscillation. The frequency spectrum of this signal will be dominated by oscillations at the base frequency and the first harmonics. Nevertheless, higher harmonics will rapidly appear not only due to contributions from the Bessel functions in Equation 16, but also from the anharmonicity of the potential. Figure 5 shows the expected detector signal alongside the Fourier spectrum for the same tip parameters as before with an anharmonicity ε = 2 × 10^8^ m^−1^ s^−2^, an oscillation amplitude *A* = 2σ*_x_* and a detection position *x*_0_ = σ*_x_*. The signals are shown including (blue lines) and neglecting (red line) particle collisions. For comparison, also the harmonic results (black line) are shown. The oscillation of the tip and the damping due to dephasing are clearly visible in the anharmonic data. As expected, the damping is strongest for the data including particle collisions.

## Experiments

Our experimental setup is based on a cold-atom apparatus, the same as that used for the first cold-atom scanning probe microscope [29,52]. It uses standard cooling and trapping techniques to generate cold-atom tips of ^87^Rb atoms in an ultrahigh vacuum environment [53]. The trapping and manipulation of the cold-atom tip is achieved via a magnetic microchip, holding a variety of micrometer-sized current conductors [54]. They produce magnetic trapping potentials that hold the cold-atom tip close to the chip surface. Tuning the microchip currents, not only the shape but also position and velocity of the cold-atom tip and the underlying potential can be precisely controlled.

**Figure 6:**
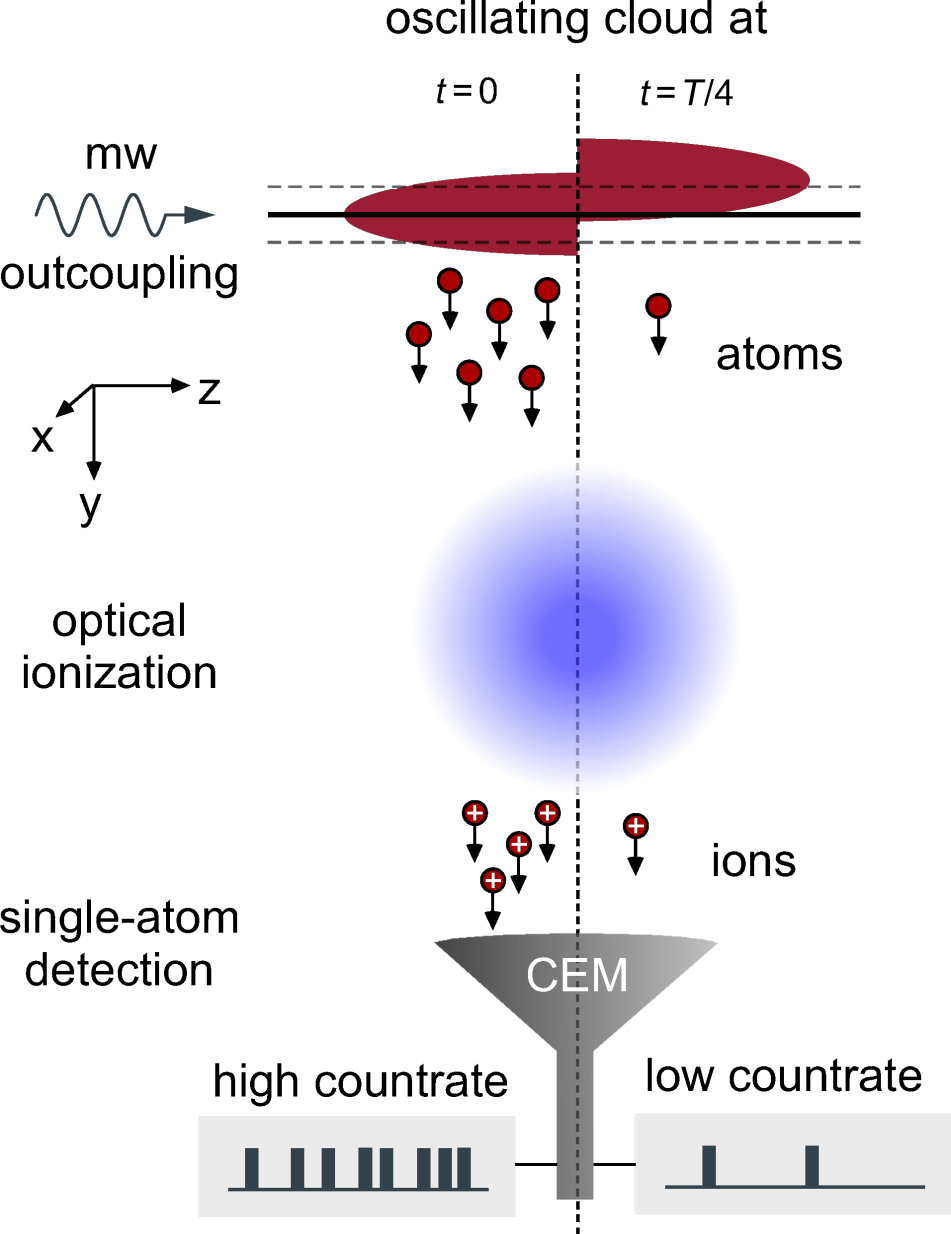
Detecting cloud dynamics. Using microwave radiation, individual atoms are outcoupled from the cold-atom tip. The outcoupling is strongly localized to the region of resonant microwave coupling (resonance sheet), typically shaped flat (black solid line) and tunable via the microwave frequency (dashed solid lines). The outcoupling rate is proportional to the atomic density within the resonance sheet and the total microwave power [51]. Depending on the cloud dynamics, this overlap changes in time, causing a time varying outcoupling rate (left and right half-image). The outcoupled atoms leave the trap and become ionized via a three-photon ionization process with lasers at 778 nm and 1064 nm. The resulting ion beam is captured and guided by ion optics [55] (not shown) and finally detected by a channel electron multiplier (CEM) [56–57]. Individual ions are detected with temporal resolution of 8 ns, allowing for the real-time monitoring of the outcoupling rate and the cloud dynamics.

The single-atom-detection scheme for measuring the tip dynamics is illustrated in Figure 6.

The technique is based on microwave outcoupling of the trapped atoms [51] with subsequent photoionization and ion detection [58]. The microwave couples atoms from the trapped 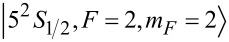 to the nontrapped 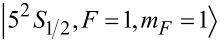 state. Due to Zeeman splitting in presence of the trapping field, the transition frequency between these states is detuned to the zero-field resonance at 6.8 GHz. Depending on the microwave frequency, the outcoupling position can thus be tuned across the cloud. Each value of the microwave frequency addresses atoms at a specific magnetic field amplitude, defining closed resonance surfaces for the microwave outcoupling. For harmonic potentials, these resonance surfaces are elliptically shaped. However, due to gravity, the cold-atom tip is displaced from the magnetic trap center and the resonance shells can be approximated plane [51]. The outcoupled atoms leave the trap and become ionized via a three-photon ionization process. The resulting ion beam is captured and guided by an ion optics [55] and finally detected by a channel electron multiplier (CEM) [56–57], yielding single-atom resolution. While individual events are detected with 8 ns resolution, the CEM saturates at count rates of about 1 MHz. This limits the maximal observable tip frequency to within this regime. However, in our specific experimental realization, the maximal observable tip frequency is at about 1 kHz, due to technical limitations in the ionization process.

For the experiments shown below, we prepare cold-atom tips in a potential, which in harmonic approximation is cigar shaped and characterized by the trap frequencies ω*_x/y/z_* = 2π × 85/70/16 Hz. The cold-atom tip consists of about *N* = 6 × 10^5^ atoms and has a temperature of *T* = 300 nK. All these parameters are deduced from standard absorption imaging [50].

## Results and Discussion

### Cold-atom tip at rest

**Figure 7:**
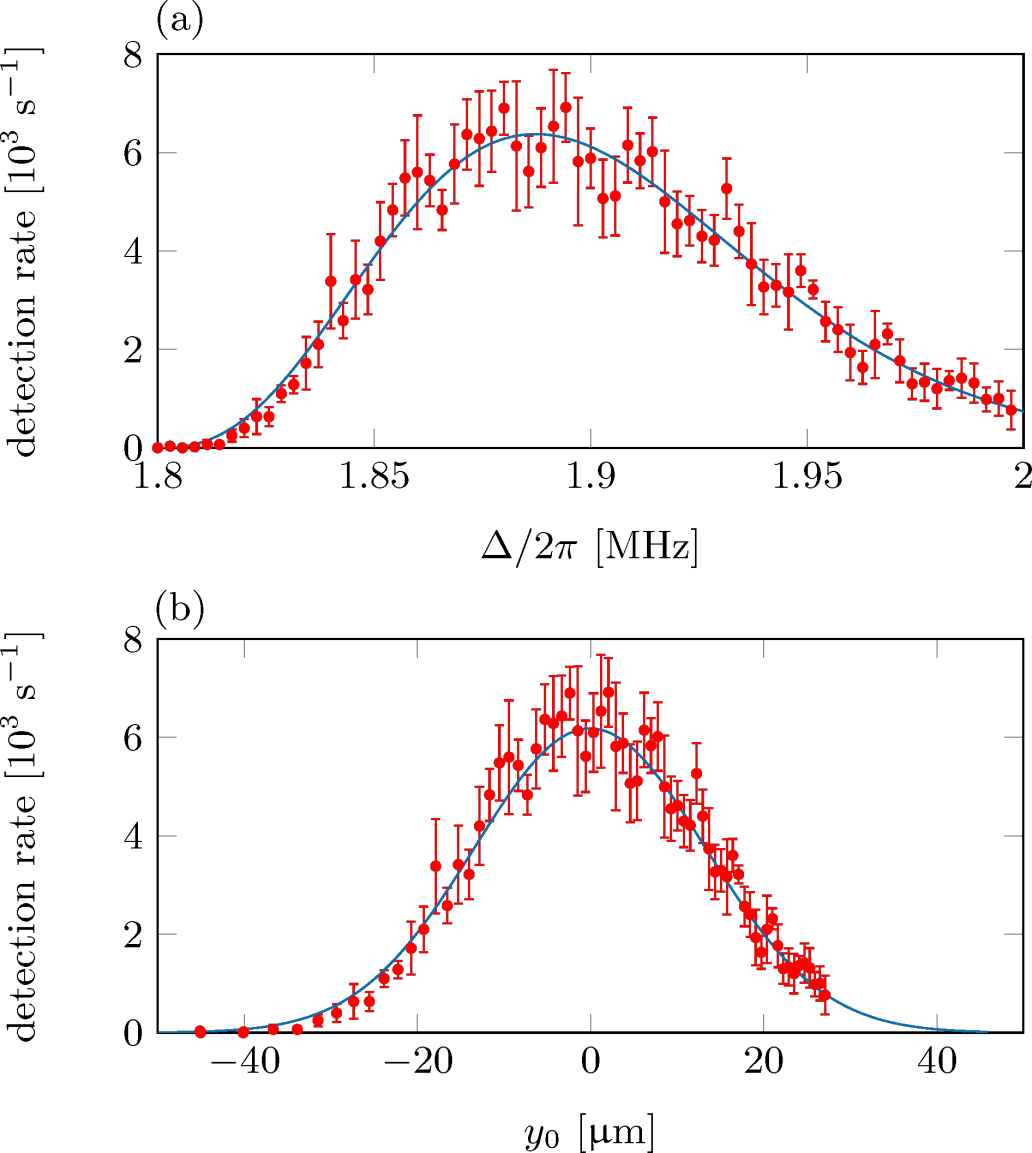
(a) Detection rate as function of the microwave detuning (spectral response), averaged over three measurements. For each measurement the microwave has been swept with a rate of 286 kHz/s, while monitoring the corresponding ionization rate. The microwave detuning is shown with respect to the zero-field transition frequency of ≈6.8 GHz between the rubidium hyperfine ground states. Each microwave frequency addresses a specific outcoupling plane at position *y*_0_. (b) Detection rate as function of *y*_0_.

We start our analysis by measuring the outcoupling rate for a cold-atom tip at rest. Therefore, we irradiate a microwave (MW) for about 1 s and measure the number of outcoupled atoms. Repeating the experiment several times yields the spectral response of the cold-atom tip. Figure 7a shows the measured ion detection rate as function of the microwave detuning Δ with respect to the zero-field transition frequency. Thereby, each microwave frequency can be connected to a specific position *y*_0_ of the resonance sheet [51], yielding a position dependent response function, as shown in Figure 7b. This function basically shows the density distribution of the cold-atom tip as expected for a cold cloud of 300 nK.

A full theoretical description of the expected response function has been given in [51] and is used here to verify the experimentally measured microwave response. The blue lines in Figure 7 show the results of this ab initio theoretical calculation as expected for our experimental parameters. They show excellent agreement with the measurements.

### Oscillating cold-atom tip

Knowing the spectral response of the cold-atom tip, we investigate tip oscillations and their corresponding detection signal. Therefore, we initiate precise center-of-mass oscillations in the direction of gravity (*y*-direction), by displacing the magnetic trap non-adiabatically, i.e., on a timescale much faster than the corresponding trap frequency. As measured with absorption imaging, the oscillation amplitude is *A* = 1.33σ*_x_* = 16.2 μm. Using our single-atom detection scheme the resulting dynamics of the cold-atom tip can be monitored in situ and real time. To transform the measured timestamps of the ion detection events to a time-discrete signal, we bin the timestamps in bins of 1 ms width. To allow for a detailed comparison of the experimental data to the numerical calculations, which do not include particle losses and microwave-induced dipole potentials, we intentionally chose low outcoupling rates. To remain a good signal-to-noise ratio, all data have thus been averaged over 50 experimental cycles. At higher outcoupling rates, tip oscillations can be detected in a single run.

**Figure 8:**
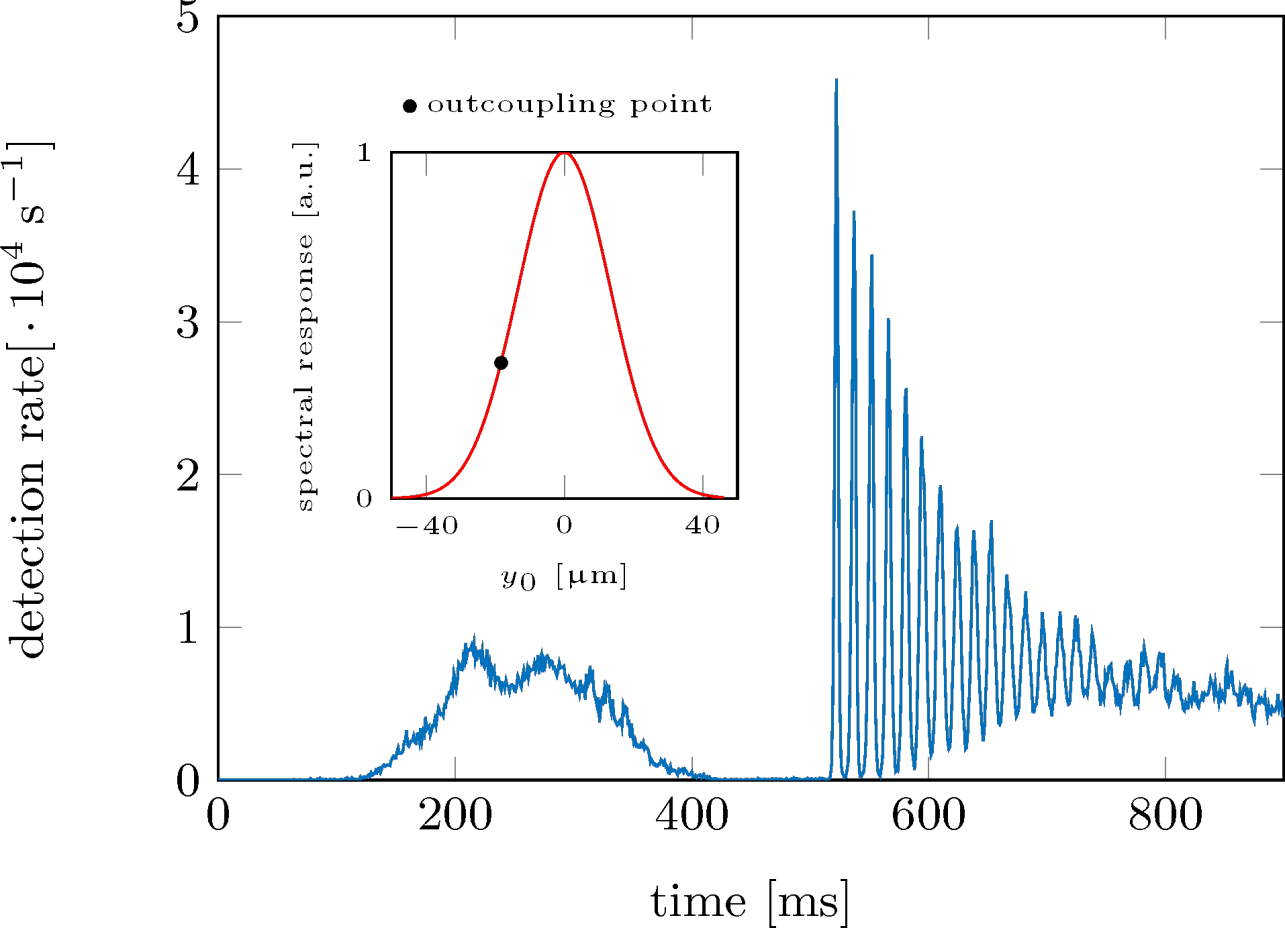
Ion signal during and after the excitation of the cold-atom tip to a center-of-mass oscillation.

Figure 8 shows an example of the measured ion signal as averaged over 50 experimental runs with the outcoupling position tuned to *y*_0_ = −18.3 μm (Δ = 2π × 1.84 MHz). The obtained signal nicely represents the experimental cycle. In the first 100 ms the ionization lasers were turned on, followed by the microwave radiation within the next 100 ms. Starting at *t* = 300 ms the cold-atom tip is first displaced adiabatically within 200 ms by an amplitude of 53 μm. Tip oscillations are now excited by moving the magnetic trap back to its starting position non-adiabatically on a timescale of 12 ms. After this excitation process, the ion count rate shows clear oscillations at the trap’s base frequency. As expected, these oscillations show a clear damping.

**Figure 9:**
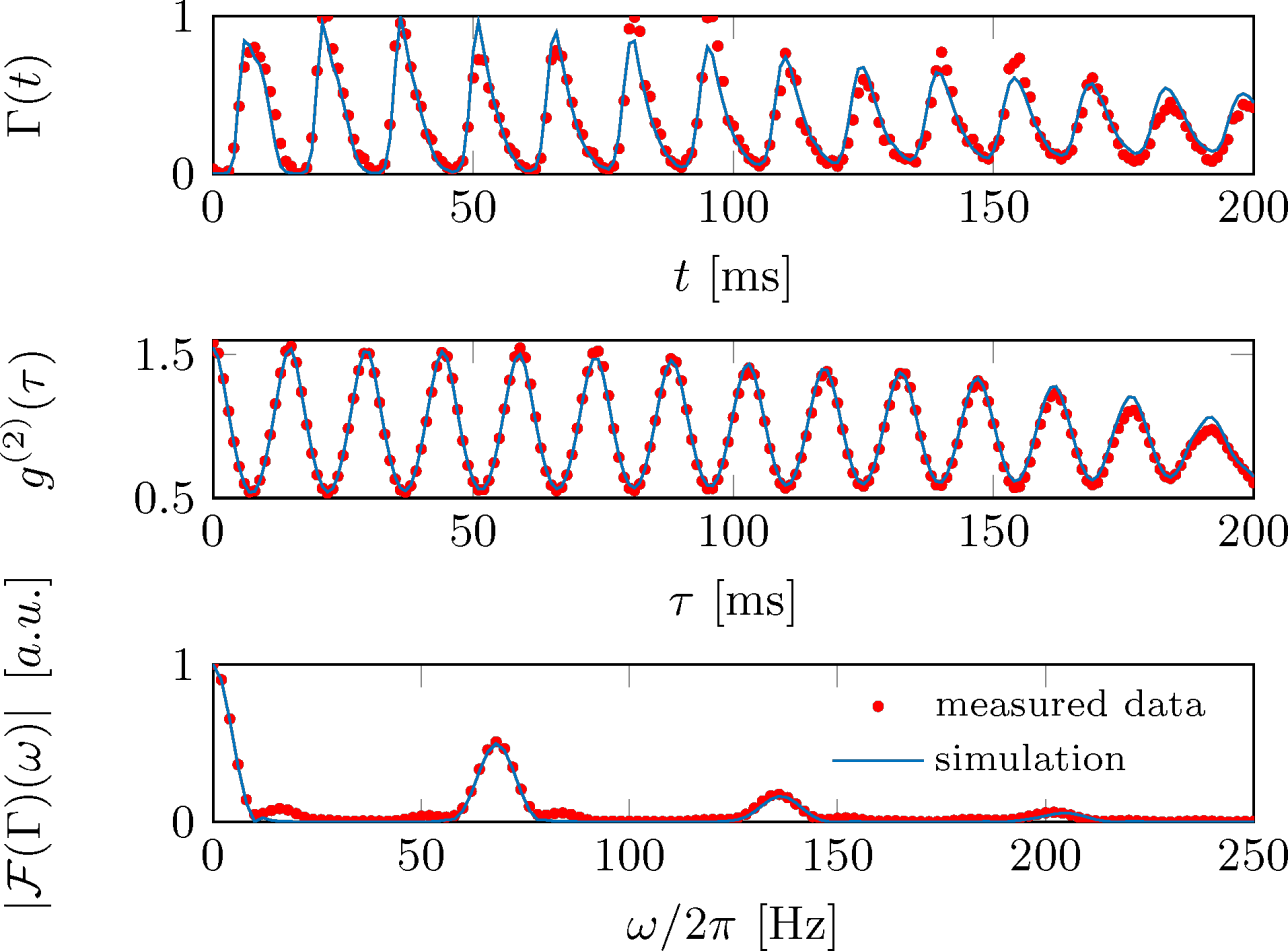
Comparison of the measured (red dots) and simulated (blue lines) data for the detection signal, the normalized autocorrelation function and the Fourier transform. The outcoupling position has been fixed at *y*_0_ = 32.6 μm (Δ = 2π × 2.03 MHz).

For further data analysis, we restrict the signal to the pure oscillation region starting at *t* = 512 ms. In addition to the binned detector signal, we also calculate the Fourier transform and the autocorrelation function as described in the methods section. These calculations are done on the single particle events rather than the binned detector signal to improve the signal-to-noise ratio. Figure 9 shows an example of the binned detector signal alongside the Fourier transform and the autocorrelation function as extracted from the measurement at *y*_0_ = 32.6 μm (Δ = 2π × 2.03 MHz).

The autocorrelation function shows a nicely damped oscillation which can be used to extract the tip’s oscillation frequency. The same information can be obtained from the Fourier transform.

Compared to the detector signal, we find the autocorrelation function to be generally better suited for extracting the oscillation frequency and amplitude, as it shows less noise. This stems from the fact that the autocorrelation function is based on a histogram of *N*^2^ particle pair distances (see methods section), whereas the detector signal is based on the *N* detection events. In addition, frequency components with small amplitude are strongly suppressed in the correlation analysis. This can be seen when using a generalized form of the detector signal from Equation 16

[18]
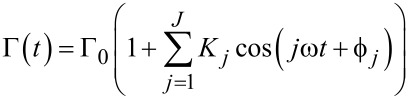


with the coefficients *K_j_* depending on the specific oscillation parameters and outcoupling position. The normalized autocorrelation function then reads

[19]
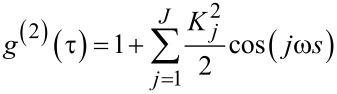


with the amplitude of the *j*th frequency component given by 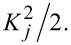 Weak frequency components are thus suppressed quadratically as compared to the original detector signal. Therefore, the autocorrelation function will typically show a clear single frequency oscillation. However, the phase information 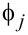 is lost in the correlation analysis.

### Signal-to-noise ratio

**Figure 10:**
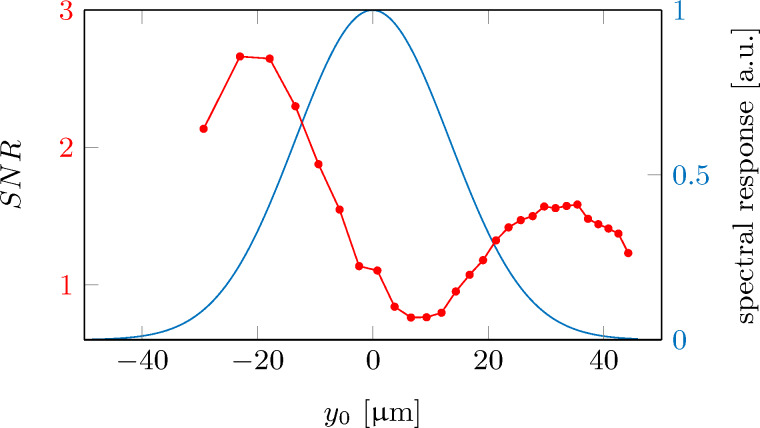
Signal-to-noise ratio (red points) as measured for different outcoupling positions alongside the atomic response function (blue line).

As described earlier, the detection signal strongly depends on the outcoupling position. For an application-orientated assignment of a cold-atom tip, the signal-to-noise ratio should thus be optimized. Therefore, we take a series of oscillation measurements (*N* = 6 × 10^5^, *T* = 300 nK, *A* = 16.2 μm) for outcoupling positions ranging over the whole cloud extension. From the corresponding autocorrelation of the detection signal we find that besides damping, the main frequency component is given by the trap’s base frequency at 70 Hz. Following Equation 19 we extract the oscillation amplitude *K*_1_ and calculate a signal-to-noise ratio where SNR = Γ_0_*K*_1_/ΔΓ_0_ with Γ_0_ = 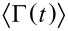 and ΔΓ_0_ is the averaged error bar of the 50 oscillation measurements at each outcoupling position. The result is shown in Figure 10 alongside the spectral response function.

As expected, the best signal-to-noise ratio is achieved at the slope of the tip response function. Here, a small oscillation amplitude leads to a large and stable signal modulation. The outcoupling at the maximum of the spectral response should typically lead to a minimum in the signal-to-noise ratio. However, the measurement shows that this minimum position is slightly displaced from the maximum of the spectral response, which is likely due to the trap’s anharmonicity and particle collisions.

### Comparing experiment to theory

To verify that the damping of the experimentally observed tip oscillation is solely due to the anharmonic component of the trap potential, we performed a full numerical simulation of the ultracold cloud dynamics and the microwave outcoupling process. Thereby, we tried to model the physical reality as accurately as possible. This includes particle interactions, as well as a realistic model of the total external potential. Moreover, the tip oscillation is excited similar to within the experiment, along one of the trap’s principal axes and including particle collisions. In addition, we use a more elaborate implementation of our detection scheme. The basic principles of the numerical simulation, the tip potential and the detection scheme are outlined in the methods section.

Figure 9a shows a comparison of the simulated detection signal and the measurement for outcoupling at *y*_0_ = 32.6 μm (Δ = 2π × 2.03 MHz). To account for the finite detection efficiency of our ion detector [59], both signals are normalized to their maximum values. The corresponding autocorrelation functions and Fourier spectra are shown in Figure 9b,c. The simulations show almost perfect agreement with the experiment, including the relative strength of the spectral lines. As the outcoupling point is positioned at the edge of the spectral response, the main frequency components are given by the trap’s base frequency ω*_y_* = 2π × 70 Hz and its first harmonic. However, the Fourier analysis reveals some minor deviations between simulations and measurement, as the experimental data show minor contributions also at the other trap frequencies ω*_x/z_* = 2π × 85/16 Hz. This is due to an imperfect excitation in the experiment, where the cloud is not oscillating exactly along the trap’s principal axis.

**Figure 11:**
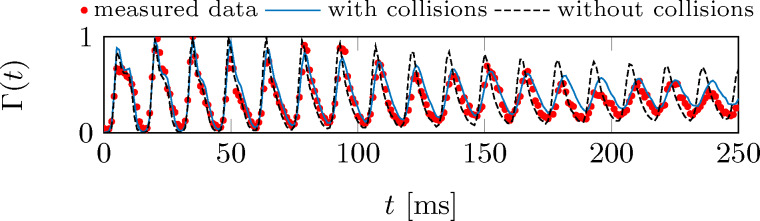
Comparison of the measured outcoupling rate (red dots) and the simulated rates, as derived from a full numerical simulation including (blue line) and excluding (black line) particle collisions. The outcoupling rates are normalized and derived at an outcoupling position *y*_0_ = 22.7 μm (Δ = 2π × 1.98 MHz).

The simulation also reproduces the correct damping of the tip oscillation. As the simulations do not include any frictional effects, we therefore conclude that the observed damping is only due to the dephasing of the cold-atom tip in the anharmonic potential and due to particle collisions. To separate both effects, Figure 11 shows experimental data for outcoupling at *y*_0_ = 22.7 μm (Δ = 2π × 1.98 MHz), as compared to theoretical simulations with and without particle collisions taken into account.

Good agreement between theory and experiment is only found when particle collisions are included. Without collisions, the damping is less pronounced and does not match the experimental data.

## Conclusion

In conclusion, we have investigated the dynamics of an oscillating cold-atom tip in an anharmonic potential and demonstrated a new technique for monitoring the tip oscillation in situ and real time. The oscillation frequencies and amplitudes can be easily extracted from the corresponding Fourier spectra and autocorrelation functions. The method will lead to tremendous improvements in the new field of cold-atom scanning probe microscopy by increasing the measurement speed by several orders of magnitude. Moreover, a full understanding of the tip dynamics will be essential for future applications of cold-atom scanning probe microscopy. The realization of precision force spectroscopy by reconstructing the trap anharmonicity from the oscillation data will be on the forefront of upcoming research. Although similar methods exist in conventional atomic force microscopy, our finding will be essential for describing the dynamics of the cold-atom tip, as it does not behave like a solid object. Our simulations show that dephasing effects and particle collisions must be taken into account to give a proper description of the tip motion.

## Methods

### Fourier transform and autocorrelation

Based on the single particle timestamps *t_i_* of all detection events *i* = 1…*N*, we calculate both the Fourier transform and the autocorrelation function. Doing these calculations on the single particle events rather than the binned detector signal improves the signal-to-noise ratio. With the detector signal 
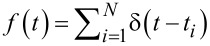
 the Fourier transform becomes

[20]
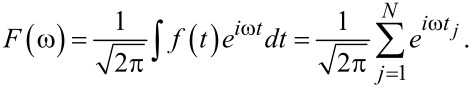


The normalized autocorrelation function is given by

[21]
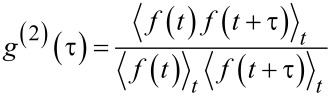


with 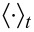 being the time average. Using our detection signal *f*(*t*), which is defined on the finite measurement time *T*, we find 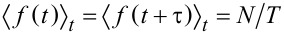 and thus

[22]
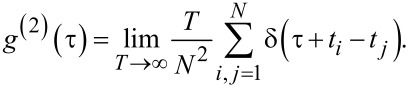


This function is transformed to a time discrete signal by appropriate binning over a correlation time interval Δτ

[23]
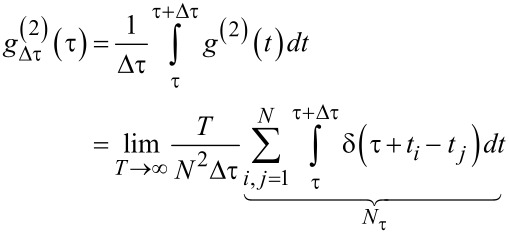


where *N*_τ_ is the number of particle pairs (*i*,*j*) with temporal separation (*t_i_* − *t_j_*) 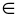 [τ,τ + Δτ]. To account for the finite measurement time, *N*_τ_ is corrected by a factor (1 − τ/*T*)^−1^ yielding

[24]
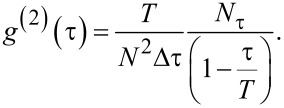


The correction factor is necessary as large time differences τ are less likely to be observed due to the finite measurement time. In practice, the autocorrelation function is found by generating a histogram of all particle pair time separations and subsequent normalization.

### Numerical simulation methods

The dynamics of the cold-atom tip is simulated via a particle simulation, which is based on solving the equation of motion for each individual particle. Therefore, we either use analytic solutions (1D harmonic simulations) or a Runge–Kutta method. Depending on the simulation complexity, we reduce the number of particles by simulating a smaller number of test particles [60]. To consider ultracold collisions (interactions) between the atoms, we implemented a direct simulation Monte Carlo (DSMC) method [60–61]. The DSMC method takes s-wave collisions of the indistinguishable ^87^Rb atoms into account, with a scattering cross section 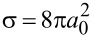 and a s-wave scattering length *a*_0_ = 5.7 nm. The concept of the DSMC method is to decouple the atomic motion and the collisions for timescales considerably smaller than the averaged time between two collision of an atom [62–63]. To include the microwave outcoupling into the simulation, we refer to a lattice simulation by describing the particle density on a lattice. The transfer to the lattice description is achieved using the cloud-in-cell algorithm [64]. Using this method, we get a numerical representation of the particle density for every point of the defined discrete time vector.

### Model of the total external potential

The main contribution to the trapping potential results from the magnetic field distribution 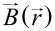 yielding a potential energy 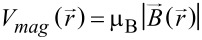 with μ_B_ being the Bohr magneton. It is produced from field generating wires and coils inside our vacuum chamber, which are partially implemented to a microchip surface. A detailed description of the microchip geometry and the other field generating elements can be found in [54] and [65]. For the experiments described here, the magnetic trap is generated from two parallel wires (QP2 wire and compression wire), which are oriented along *z* and separated by Δ*y* = 1.9 mm. The QP2 wire is implemented on the microchip surface and the compression wire is embedded into the microchip holder. With counter-propagating currents, they produce a linear quadrupole field above the chip surface with atomic confinement in radial (*x*,*y*)-direction. Trapping in *z*-direction is achieved by superpositioning an inhomogeneous field along *z*. It is produced by a set of chip wires (transport wires) oriented along *z* and implemented on the back side of the microchip. Additional homogeneous fields along *y* and *z* are applied via magnetic coils far away from the trapping region.

Including gravity, we model the total external potential via

[25]
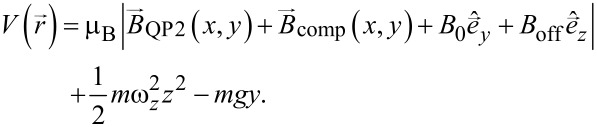


Here, we include the field of the QP2 and compression wire explicitly using an infinite long wire approximation. The axial confinement is approximated via a harmonic potential along *z*. Homogeneous fields in *y* and *z*-direction are taken into account. Using this model function, the full trap anharmonicity in the *x*,*y*-direction is reproduced.

For the numerical calculation we fix the parameters of the model function as follows: For the currents we use the experimental parameters (*I*_QP2_ = 0.85 A and *I*_comp_ = −3A). The offset field along *z* can be deduced from the spectral response measurement as described in [51], yielding *B*_off_ = 0.857 G. The remaining parameters ω*_z_* and *B*_0_ are fixed by matching the trap frequencies of the model potential (harmonic approximation at the trap center) to the trapping frequencies measured via absorption imaging.

The center-of-mass oscillation is initiated similar to the experiment by displacing the trapping potential along the *y*-axis. In the experiment this displacement is achieved by changing *B*_0_ via a pair of coils (transfer coils). For the simulations, we change the model parameter *B*_0_ in the same ratio as the current in these coils (≈12%), leading to oscillations in the radial (*x*,*y*)-directions.

### Microwave outcoupling

All simulations in the two theoretical sections are based on the simplified outcoupling theory from Equation 14. For the simulations in the experiments section, a more elaborate implementation is used. It follows from [51], with the outcoupling rate given by

[26]
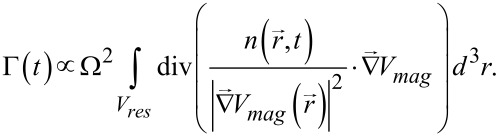


Here 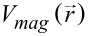 is the magnetic part of the total external potential, 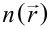 is the particle density and *V_res_* the volume enclosed by the resonance surface, which is defined via the points of resonant microwave coupling. Ω describes the microwave coupling strength (Rabi frequency), which depends on the microwave power, its polarization and the specifically coupled hyperfine states. It is calibrated independently via standard Landau–Zener sweeps [51] and assumed to be constant across the outcoupling region. Using the magnetic part of Equation 25 and the densities from the numerical particle simulation, Equation 26 can be evaluated, yielding the expected detection signal.
